# Blood Trace Element Status in Camels: A Review

**DOI:** 10.3390/ani12162116

**Published:** 2022-08-18

**Authors:** Mutassim M. Abdelrahman, Ibrahim A. Alhidary, Riyadh S. Aljumaah, Bernard Faye

**Affiliations:** 1 Department of Animal Production, College of Food and Agriculture Sciences, King Saud University, Riyadh 11451, Saudi Arabia; 2 CIRAD-ES, UMR SELMET, TA/C 112A, Campus International de Baillarguet, 34398 Montpellier, France; 3 Faculty of Biology and Biotechnology, Department of Biotechnology, Kazakh National University Al-Farabi, Almaty 050040, Kazakhstan

**Keywords:** camel, trace element, mineral deficiency, mineral toxicity, physiological variability

## Abstract

**Simple Summary:**

Trace elements are required in small quantities for all species, and their role in many biological functions is essential. Additionally, their deficiency or excess can have important consequences for metabolism and health. Although camels live in ecosystems dominated by minerals, they are also subject to such imbalances. To investigate the trace element status of camels, as for other animals, blood sampling is preferred. The present paper gives a large overview of the values observed in camel blood samples under different physiological and health conditions, as well as some data regarding the symptoms in deficiency or excess situations. The main trace elements involved are copper, zinc, iron, selenium, manganese, cobalt, iodine, fluorine and molybdenum. In addition, some data regarding bromide and nickel, due to their specific mention in the literature, are included. Although the status of sulfur is regarded as intermediary between the main and trace elements, it is added due to its role in polluted areas. Finally, some specificities of camel trace elements are discussed.

**Abstract:**

Trace minerals play an important role in animal health and productivity. They are involved also in many physiological activities, and their deficiency causes a variety of pathological problems and metabolic defects, reducing consequently the animal productivity. The demand for animal products in semi-arid areas is rapidly increasing, and the supply is still below the required level, partially due to low animal productivity. Camels (*Camelus dromedarius* and *Camelus bactrianus*) are considered one of the main sources of healthy, high-quality meat and milk for human consumption within most of the countries in the semi-arid regions. Despite their efficient adaptation to their environment, camels can suffer from the growth retardation of newborns, low feed efficiency, anemia, poor fertility, poor reproduction and many other metabolic disorders. It is well known that trace mineral deficiencies and trace mineral toxicities can influence camels’ production and reproductive efficiency, as well as many aspects of their growth and metabolism. Evaluating the trace minerals status of camels and their variability is an obvious step toward improving camels’ productivity and health. Thus, the present article reviews the data regarding the status of trace minerals (copper, zinc, iron, selenium, manganese, cobalt, iodine, fluorine, molybdenum, sulfur, bromide and nickel) in camel blood and their physiological variability, with a focus on their deficiency and toxicity effects.

## 1. Introduction

The arid lands of Africa and Asia are under pressure due to global warming, which is affecting the rangelands’ productivity and the feed resources for livestock [1], especially the camel, which is the most adapted domestic animal to such ecosystems. As a result of these changes, the trend for camel farmers is to modify their production system based on herd mobility to primarily settled, semi-intensive systems [2,3]. For the livestock remaining under extensive systems, wide seasonal variations affect the quality and quantity of the feedstuffs and consequently the nutritional status of the grazing animals, as well as their health and productivity directly and indirectly [4,5,6,7,8,9,10]. Under more intensive systems, camel feeding is progressively becoming dependent on supplements as a means of meeting the nutrient requirements [11]. In this context, the feeding system is changing from a highly diversified diet (with high variability in nutritive value and grazed ecosystems) to a standard diet (typically alfalfa + occasionally barley + concentrates) [3]. Such diets do not necessarily cover the nutrient requirements, including trace minerals [12], leading to low growth or low milk productivity [13,14].

Trace minerals contribute to the camels’ health and productivity, especially when they become a limiting factor in the diet [15]. In herbivores, trace minerals play a pivotal role in many physiological activities, and their deficiency causes a variety of pathological problems and metabolic disorders [16,17,18,19], including in camels [20]. Infertility, non-infectious abortion, anemia and metabolic diseases are some of the main clinical signs of deficiencies and abnormalities [21,22]. A few scientific studies have shown some evidence of the sensitivity of camels to trace mineral disorders, resulting in either deficiency or toxicity in the same way as in other ruminants [23]. Faye et al. [24], Faye and Bengoumi [23] and Liu et al. [25] have reported several incidences of clinical mineral deficiencies in camels being underestimated because the signs of subclinical deficiencies may remain undetected for long periods. Regarding toxicity, the evidence is even rarer, although some cases of selenosis have been described [26], as well as fluorosis [27].

Under field conditions, the trace mineral status of animals in terms of deficiency, adequate supply or excess are difficult to assess. Different substrates such as urine, feces, and hair can be convenient for investigating the mineral status of animals [15], but blood remains the most common biological material used in practice to detect a deficiency or toxicity under field conditions. A short overview regarding the metabolic functions of trace elements is given below, but the present review is mainly focused on the outcomes of the blood data available from camels and the limits of interpretation. Moreover, the specificity of camels compared to other livestock is discussed at the end.

## 2. General Overview of Trace Minerals Functions

Factors such as the level of nutrition, mineral availability and mineral utilization affect the production and reproductive ability of both males and females in ruminant animals [19]. The effect of a specific mineral on metabolism can be observed in the four stages that characterize the development of deficiency. The first stage, the initial depletion, is restricted to changes in the metabolism of the element itself (the adjustment of absorption and upregulation of carriers). The second stage, the compensated metabolic phase, is characterized by changes in the element-dependent function; this can be compensated for by another independent system unless stress is imposed. The third stage, metabolic deficiency, involves changes in the major metabolic pathways (nucleic acids, proteins, carbohydrates, and fats). The fourth stage, clinical deficiency, involves clinical symptoms, disease and eventually death [28].

Because the diversity of proteins and enzymes containing Zn, Cu, Mn, Co, I and Se, these trace minerals are essential for a wide variety of physiological processes regulating growth, production, reproduction, and health. Deficiencies in these nutrients consequently lead to reduce performance, and dairy cattle diets are therefore formulated with trace mineral supplements to prevent these deficiencies as mentioned in many papers [29,30,31,32,33,34,35,36,37,38,39,40,41]. For example, cobalt is a main component of vitamin B12 (cyanocobalamin) and plays an important role in microorganisms of the rumen [42] while iodine is involved in the synthesis of thyroid hormones [43].

One of the most important roles of trace elements is their contribution to the antioxidative function. This role is particularly efficient in stressful situations [30,32,44]. Oxidation is a normal process that produces free radicals, and the antioxidant system is activated to neutralize these free radicals before they cause cellular damage. Zn, Cu and Mn are integral components of this system due to their presence in superoxide dismutase (SOD), which reduces the free radical superoxide to hydrogen peroxide. Selenium is a component of glutathione peroxidase (GSHpx), which then converts hydrogen peroxide into water. Oxidative stress is an imbalance between antioxidants and oxidants in favor of the latter [45].

In a healthy animal, the antioxidant system reduces free radicals as several agents are produced to prevent them from damaging cells and metabolites. However, under severe stress, the rate of free radical production can exceed the rate of free radical neutralization by the antioxidant system, and this can lead to oxidative damage to the lipids, carbohydrates and proteins within cells [44]. Examples of such times of oxidative stress include calving, infection and heat stress [44,46]. Higher-producing cows have also been shown to have greater concentrations of oxidatively damaged lipids than lower-producing cows [47].

During late gestation, the immune function is weakened, and dairy cows have a decreased capacity to stay in good health [48]. Oxidative stress, non-esterified fatty acids, ketones, negative energy balance and inadequate calcium status are the main factors believed to be responsible for this immunosuppression [49]. The health disorders associated with oxidative stress include mastitis, retained fetal membranes and udder edema. The trace minerals with an antioxidant function include Se, Cu, Zn, Mn and Fe. Some diets have a role in directly quenching free radicals [50]. Minimizing health disorders during late gestation is economically advantageous because diseases are costly, not only in terms of treatment costs, but also because of the subsequent decreases in lactation performance and reproduction, the increased risk for additional health disorders and the decreased market value and productive life [51].

Thus, a deficit of minerals could be associated with different situations of stress, notably during seasonal changes, leading for example to a decrease in serum concentrations of copper (Cu), Co and Zn, as it has been reported in grazing dairy cattle in western Sudan during the late dry season [52]. Similar seasonal changes in mineral profiles (Cu, Zn, Mn and Co) were detected in the serum samples of grazing Jordanian sheep [53] and cattle [54]. Another study on the mineral and vitamin status of sheep in Syria, Jordan and Turkey, in which blood samples were collected from 18 sites, indicated a decrease in Cu and Zn levels at the end of the winter season [55].

## 3. Trace Mineral Status in Camel Blood

The nutritional status of camels has recently received significant attention, particularly in developing countries that depend on their products (meat, milk) as a main source of nutrients for human consumption. Recently, there has been a greater focus because of the putative role of a variety of camel products in the treatment of different diseases [56,57]. However, the requirement of camels for key nutrients such as trace minerals has received little attention compared to other farm animals [58]. Most of the available data are limited to few countries involved in rearing camels, such as Morocco [59,60], Sudan [61], Ethiopia [62], Djibouti [63], Saudi Arabia [64], the United Arab Emirates [65], Oman [66], Iran [67], India [68] and China [69]. Although Sahelian countries have the largest camel populations at the global level [70], no investigation has been achieved on the trace element status of camels in this region. In the review by Schillhorn van Veen and Loeffler [71] in 1990, focused on mineral deficiencies in ruminants living in Sub-Saharan countries, camels were not included.

In the following part, normal levels of trace elements in the blood and serum and deficiency or toxicity cases are reported. Usually, trace elements are determined in the serum (when extracted after coagulation) or plasma (when extracted before coagulation), and more rarely in the whole blood (selenium, iron). Some differences were reported in the concentrations between serum and plasma, for example for copper [72]. Thus, comparisons with the different results in the literature are not easy. The type of blood substrate is sometimes specified when it appeared convenient for our purpose.

However, a diagnosis of deficiency or toxicity cannot be limited to blood values in a limited number of animals. To achieve a clear diagnosis, some other considerations must be taken into account: (i) blood (or plasma and serum) is not the only substrate for the assessment of the nutritional status of trace elements; other substrates such as milk, urine, liver, hair or other organs can be considered, but they are not generally used in routine investigations; (ii) blood results mainly draw a collective interest because deficient or excess status can be accepted only if a majority of the herd presents values below or above the threshold that is generally admitted; (iii) the environmental conditions, the interactions between elements, the health status and the diet components are essential for a clear interpretation of the mineral status of the animals, but those parameters are often lacking in the publications; (iv) an individual diagnosis is only efficient in the case of clinical signs of deficiency or toxicity. Thus, considering that blood sampling generally reflects the trace element intake, and because blood is the main investigated substrate for probing the mineral status of animals, only data regarding plasma or serum minerals are reviewed in the present paper. The other substrates can be used also, but have limited (milk) or debatable (hair) interest or require more complicated techniques (biopsy, urine collection) or animal euthanization (muscle and organs). For more information regarding the other substrates, the readers can refer to the book by Faye and Bengoumi [15].

### 3.1. Copper

#### 3.1.1. Normal Values

According to the existing literature, the normal levels of copper in camel plasma are similar to those in cows, being within the range of 70–120 µg/100 mL [15]. In their monitoring of 14,237 racing camels in the United Arab Emirates, Wernery et al. [73] reported a mean plasma copper value of 71 ± 17 µg/100 mL. In different environments, between-species comparisons have been reported, showing a trend of higher copper values in camel plasma (Table 1), probably due to the diet of the camels, which includes higher proportions of trees and bushes [74].

#### 3.1.2. Variation Factors

Globally, there were no sex-dependent differences in the copper status of camels. Even when significant statistical differences were observed [81], the values were close in both sexes without biological significance, at 61.9 and 56.7 µg/100 mL for females and males, respectively. However, in females, the pregnancy status is linked to some changes in plasma copper values, with a decrease at the end of gestation, probably linked to active transfer to the fetus [82,83]. The difference between pregnant and lactating camels was more often investigated, leading to contradictory results [65,83,84]. A significant increase was also observed throughout the lactation ranging from 96 ± 3 in the early period to 130 ± 2 µg/100 mL in the last period [85]. Similar contradictory results were reported regarding the age effect [61,84,86,87]. Few studies exist describing plasma copper changes in camel calves post-partum. According to Hussein et al. [88], cupremia regularly increases from birth (50 µg/100 mL) to 5 months of age, reaching a plateau in the range of 63–76 µg/100 mL at up to one year. The breed variability was also investigated in Saudi Arabia, where significant differences were reported between Majaheem (black coat camel) and Waddha (white coat camel) camels, with mean values for plasma copper of 71.2 and 57.5 µg/100 mL respectively [89]. However, for other factors, such observations are not indisputable since other authors have found no breed differences [16].

In fact, the physiological factors seem to have a weak impact on the copper status compared to the nutritional factors. Indeed, several studies have emphasized the significant impact of copper supplementation, whether as copper sulfate in salt, bolus or enriched concentrates [12,90,91,92,93]. The impact of water restriction was also investigated by several authors, showing an increase in plasma copper in dehydrated animals in concert with the decrease in blood volume [94]. The changes in blood copper values linked to health status could occur mainly because of the inflammation process, which provokes hypercupremia, as observed in other species. However, this aspect was not investigated in camels, and in reverse a decrease in the copper concentration in the blood was reported in cases of testicular degenerative injury [95], anemic camels [96] or mange [97], but no effect of mastitis or internal parasites was revealed [98,99]. In camels hospitalized in several vet clinics from Egypt, Hassan et al. [100] did not find any difference between healthy and sick camels, regardless of the disease (respectively 92.7 ± 2.5 and 92.9 ± 0.5 µg/100 mL).

#### 3.1.3. Copper Deficiency and Toxicity

In general, serum copper levels below 50 μg/100 mL can be considered deficient, although a safe diagnosis of deficiency cannot be done at the individual level, but rather at herd level due to the large individual variability already highlighted here [15,75]. Abu Damir et al. [12] reported a high incidence of hypocupremia in breeding camels in the UAE due to low dietary copper. Low concentrations were also reported in grazing camels from Pakistan due to low copper levels in forages [101]. However, the lowest plasma copper levels recorded in the literature were observed in Djibouti in dromedaries grazing in mangrove trees [102], with concentrations reaching only 5 μg/100 mL, although no clinical symptoms of copper deficiency appeared. In Chinese Bactrian camel, ataxic disease, similar to ovine “sway-back”, associated with low copper values in the blood (mean of 28 µg/100 mL) was reported [24]. Low copper values (28-31 µg/100 mL) were also reported in different regions of Sudan [57]. In the Rift Valley, Faye et al. [80] reported cases of secondary copper deficiencies due to the interactions between copper, molybdenum and sulfur, which led to the synthesis of copper thiomolybdate, a compound practically unassimilable in the gastro-intestinal tract of the herbivores [103]. However, the differences in feeding behavior led to a more severe effect on sheep and cattle than in camels [80].

Copper toxicity in natural conditions was not reported, but experimental intoxication was achieved by Abu Damir et al. [104], provoking anoxia, the regurgitation of the gut contents, teeth gnashing, diarrhea and the lateral decubitus position before death within 8 days of injecting 200 mg copper.

### 3.2. Zinc

#### 3.2.1. Normal Values

Although normal zincemia in ruminants is similar to cupremia (i.e., between 70 and 120 µg/100 mL), this reference range is highly questionable for camels. Indeed, many references regarding healthy camels reported plasma zinc values below 50 µg/100 mL [15]. Moreover, contamination during sampling is highly probable. In the case of hemolysis (despite camel red cells being resistant), zinc present in high quantities in erythrocytes can artificially increase its concentration in the plasma or serum. In the literature, the values can vary from less than 25 ± 15.8 µg/100 mL on average (for example in the study by Mustafa et al. [105] in Sudan) up to 186.7 ± 15.1 µg/100 mL (in the study by Parekar et al. [106] in India), but recent data have revealed that camels are able to maintain Zn in the serum at a level less than 60 μg/dL, even with oral zinc supplementation, in contrast to copper [107,108,109]. Indeed, zinc homeostasis works efficiently both in animals and humans [110]. Zinc homeostasis is based on synergistic adjustments to the zinc absorption and endogenous intestinal excretion processes. Thus, the endogenous excretion of zinc increases as soon as the zinc intake becomes excessive, and the zinc absorption increases in cases of low intake. However, the mechanisms of zinc homeostasis in camel have not been studied. Additionally, most of the comparative studies, contrary to copper, have reported lower zinc values in camels (Table 2). Compared to cows receiving a similar diet (including zinc supplementation), the plasma zinc levels appeared to be lower before, during and after oral zinc sulfate supplementation [107].

#### 3.2.2. Variation Factors

The age and sex effects were examined by several authors, but no clear trend has emerged, since contradictory observations were reported. Generally, young camels still suckling have higher zincemia than adults [65,113], but this observation was not constant [57,83]. A sex effect has not been constantly observed also. Although Seboussi et al. [65] found that the plasma zinc concentration is significantly higher in males (23.9 µg/100 mL) than in females (14.1 µg/100 mL), in most of the studies there was no difference. Zinc concentration in the plasma of non-pregnant, non-lactating camels also appeared to be higher than in pregnant and lactating camels [65,68].

In pregnant camels, zinc concentration declined at the end of gestation [20,82], for the same reason as for copper (active transfer to the late fetus [114]. Contrary to the results found by Seboussi et al. [65] in the UAE, significant differences were observed between camel breeds in Saudi Arabia [89] and in India [16], but despite the statistical significance, the biological meaning is of low interest because the values were within the normal range.

In contrast to copper, zinc supplementation seems to have a mild effect on the zinc status of the animal. Young deficient camels supplemented with trace elements did not show an improved zinc status, contrary to copper [24]. In a comparative study including camels and cows, oral zinc supplementation did not significantly increase zincemia in camel (+15%), while zinc was increased by 43% in cows [111]. This effect is reinforced when the copper supplementation is associated with zinc supplementation, because a negative interaction occurs between zinc and copper, which are in competition at the absorption sites of the intestines, as it has been shown in rats [108]. Indeed, in camels, copper supplementation led to decreased zincemia [60].

However, by using a trace element bolus with long term release, Alhidary et al. [90] were able to improve the zinc status. Moreover, all studies reporting seasonal variations emphasize the potential impact of variability in feeding resources on the zinc status of camels [83,84,115].

#### 3.2.3. Zinc Deficiency and Toxicity

Thus, due to the high variability of values reported in the literature, it is difficult to categorize the zinc deficiency in camels just based on low zincemia [116]. Moreover, contrary to copper, which increases in cases of acute inflammation, zincemia generally decreases. Consequently, some disorders, notably those affecting reproduction [18] or linked to internal parasites, can be linked to lower zincemia [93]. As zinc plays an important role in skin protection, some authors hypothesize that the low zinc status in camels could explain the sensitivity of this species to skin diseases [117]. Recently, the zinc concentrations were determined in camel skin [118] to be 115 ± 60 (for the external side) and 94 ± 82 ppm (for the internal side). Further studies should emphasize this aspect by comparing healthy animals to camels affected by skin diseases such as mange, dermatitis, ecthyma and other disorders.

To our knowledge, zinc toxicity has never been described. The zinc tolerance is high in herbivores, especially when it is orally supplemented [119]. Even in polluted environments with high zinc levels, no clinical symptoms were observed in grazing camels [120].

### 3.3. Iron

Iron is included among trace elements, but the studies on its status in herbivores are of low interest because deficiency has not been reported in animals grazing under natural conditions. The cases of anemia in relation to hemo-parasitism such as trypanosomosis, one of the most common diseases in camels, are not the direct consequence of iron deficiencies linked to the diet. Moreover, the potential diagnosis of an iron deficiency is usually based on an analysis of biochemical parameters such as hemoglobin, transferrin or ferritin, or hematological parameters such as hematocrit. In the literature, the iron values in camel varied in the range of 40–56 mg/100 mL in the whole blood, 85–120 µg/100 mL in the plasma and 100–320 µg/100 mL in the serum [15], with contradictory results regarding the physiological factor variations.

### 3.4. Selenium

Although the biological role of selenium is widely studied in farm animals, the selenium status and metabolism were relatively recently investigated in camel [121].

#### 3.4.1. Normal Values

Selenium can vary considerably according to the area due to the presence or absence of seleniferous plants [67]. Although potential intoxication with seleno-accumulator plants has been described in East Africa, seleno-deficient pastures are also observed in different countries [122]. Such environmental conditions could explain the large variability in blood selenium levels linked to the high variability in the potential intake. Moreover, the studies of selenium status involved concentrations either in the whole blood or in the plasma or serum, leading to some confusion in the interpretation, as an important part of the biological selenium is concentrated in erythrocytes [123]. In camel plasma, the normal level is around 100 ng/mL [14]. Indeed, some studies in Morocco [124], China [25], the UAE [125,126], Sudan [61] and Saudi Arabia [127] reported such mean values. However, lower concentrations of between 25 and 53 ng/mL were found by Abdelrahim [128] in Sudan, 21 ng/mL being found by Bengoumi et al. [129] in Morocco, 12.6 ng/mL by Nafizi et al. [130] in Iran and 50 ng/100 mL in healthy camels from Egypt [97]. On the other hand, higher concentrations were recorded by other authors, with 274 to 288 ng/mL being found in Chinese Bactrian camels [131] and 200 ± 90 ng/mL in dromedaries from the UAE [61]. In a large sample of Saudi camels intended for meat production, Barri and Al-Sultan [132] reported selenium concentrations in serum varying from 5.3 to 131 ng/mL. Such discrepancies could be linked to the high sensitivity of camels to Se supplementation [129], leading to great variation among the animals. For example, in a recent experiment aiming to assess the effect of selenite–triglyceride supplementation on the selenium status of dromedary camels, the values were between 40.18 and 198.79 ng/mL [133]. With 2 mg/day of Se oral supplementation, the serum value was three times higher than that of the control, at 305.9 ± 103.3 vs. 109.3 ± 33.1 ng/mL, respectively [126]. Supplementation with organic Se appeared more efficient than sodium selenite [127] and Se injection [134]. Generally, normal values between 50 and 200 ng/mL can be retained. The daily Se intake should not exceed 4–8 mg, and the dietary levels should not exceed 0.5–1 mg/kg [121].

#### 3.4.2. Variation Factors

As for the previous trace elements, the effects of sex, age and physiological status are not clear. Elrayah et al. [61] did not find any age or sex effect. For Seboussi et al. [65], the plasma selenium increased in pregnant females compared to non-pregnant or non-lactating females, while there was no difference between pregnant and lactating camels, despite the positive maternal transfer [135]. Such differences were not confirmed in cases of Se supplementation [126]. According to Seboussi et al. [65], adult males presented lower values than females (139 ± 5 vs. 229 ± 7 ng/mL, respectively), and young animals (3–4 years old) had lower values than young adults (5–7 years) but were close to those of oldest adults (>8 years), at 142 ± 43, 281 ± 7 and 129 ± 8 ng/mL, respectively. Regarding the breed effect, there was an inconsistency compared with the study by Abdelrahman et al. [136], who found a two-fold higher value in Majaheem camels (147.1 ng/mL) than in Waddha camels (73.3 ng/mL), while Seboussi et al. [65] found no genetic difference. In a camel hospital, Hassan et al. [100] reported significant lower values in hospitalized camels (29.5 ± 1.3 ng/mL) compared to healthy ones (50 ± 3 ng/mL).

#### 3.4.3. Selenium Deficiency and Toxicity

Selenium deficiencies were suspected for a long time in cases of heart failure or muscle dystrophy in both dromedaries [137] and Bactrian camels [25]. The clinical symptoms preferentially affect young animals and are generally described under the name “white muscle disease” (WMD). This disease is characterized by degenerative myocarditis and discoloration of the skeletal muscle, and is widely described in the UAE, where the available feedstuffs are seleno-deficient [65,138]. WMD can lead to sudden death. Some cases have been reported in adult camels [139]. The threshold for serum selenium for considering a deficiency is below 35 ng/mL according El-Khouly et al. [138]. However, in young adults, Al-Qarawi et al. [139] observed the appearance of clinical signs of Se deficiency only in animals with serum selenium concentrations below 5 ng/mL.

**Figure 1:**
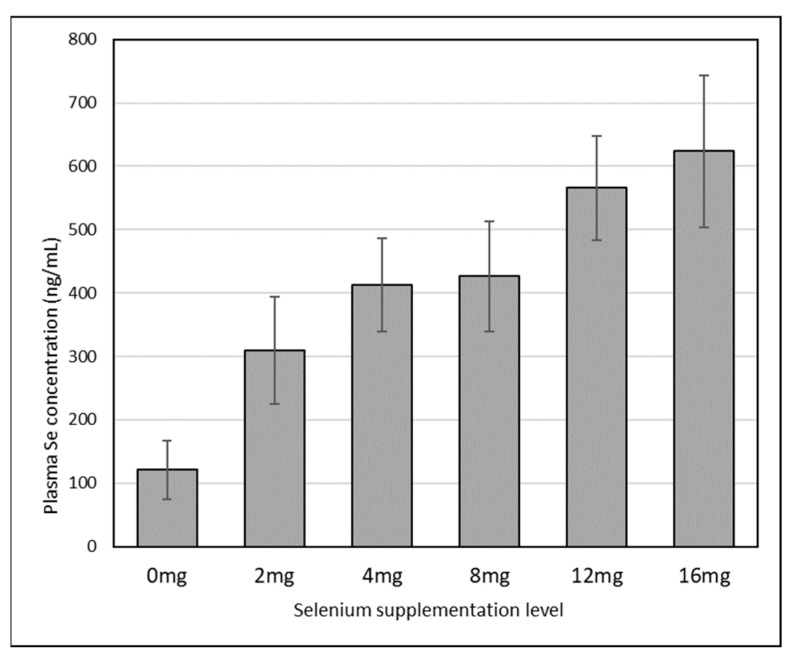
Changes in camel serum Se concentration according to the level of oral Se supplementation (reported by Faye and Seboussi, 2009 [121]).

Toxicity to selenium has never been described in natural conditions in camels. Experimental selenosis was found after oral supplementation at over 8 mg Se/day [26], provoking alopecia, diarrhea, weakness, dyspneic respiration and fissured pads. In severe intoxication (more than 16 mg/day Se supplementation), death can occur. The necropsy examination will show muscle discoloration, ascites and necrosis of the liver and kidneys. These lesions are close to those of Se deficiency, leading to the consideration that Se deficiency and Se excess could provoke the same type of cell damage [15]. Moreover, the levels of recommendation to gain a normal Se status and the higher limit to avoid intoxication seem very narrow in camels. In their study, Bengoumi et al. [129] compared camels and cows receiving the same diet (qualitatively and quantitatively) and the same Se supplementation (2 mg/day) reported a serum Se increase in cows from 33.2 ± 3.9 before supplementation to 51.1 ± 5.6 ng/mL after, while in camels it was from 20.8 ± 3.2 to 129.6 ± 30.2 ng/ML. In the meta-analysis by Faye and Seboussi [121], reporting the Se values in camel serum samples according to different levels of Se supplementation (distributed manually in date fruit), it appeared that on average the serum status reached a plateau above 4 mg/day (Figure 1). Above this value, the absorption sites of Se were saturated.

### 3.5. Manganese

Mn is not often taken in consideration in trace element monitoring in camels because the clinical interest is low [14], although the risk of deficiency is locally possible, as was reported in cattle [140]. The range of blood values in camels reported in the literature is quite wide, varying from less than 10 µg/100 mL as for other ruminants [60,75,110], to around 25 µg/100 mL [64,66,101,116,137,141], up to 160 µg/100 mL [142] and even 174 µg/100 mL [143].

Among the various factors, only seasonal changes linked to breeding status were observed [144], as well as a breed effect in Arabian camels [89].

### 3.6. Cobalt

Due to its role in the biosynthesis of cyanocobalamin (vitamin B12) in the rumen, cobalt can be a limiting factor for gluconeogenesis. A cobalt deficiency can affect the energetic metabolism, provoking a loss of appetite and growth depression, leading to severe emaciation of the animal. Nevertheless, although well described in cattle, cobalt deficiencies have not been reported in camels, probably due to the specificity of the camel diet, which contains more forage trees, which are richer in cobalt than grass [59]. However, signs of Co and vitamin B12 deficiencies were detected in camels, sheep and goats raised in the Al-Qassim region in Saudi Arabia, affecting the performance of these animals (unpublished data).

Most of the data regarding cobaltemia, which more reflects the Co intake than the Co status of the animal, originate from China and Mongolia (only Bactrian camels). The reported values were widely variable, at 3.4 to 13.2 µg/100 mL [145] with seasonal variations, 53–56 µg/100 mL in pregnant and lactating camels [25], 61 ± 12 µg/100 mL [69], 59–61 µg/100 mL [131] and 56–61 µg/100 mL [146]. In dromedaries, similar variability was reported, ranging from 0.08 µg/100 mL [75] or 0.29–0.47 µg/100 mL [67] to 11 µg/100 mL [61], and from 29 to 43 µg/100 mL according to different breeds in India [16]. No effect of physiological parameters (age, sex, lactating stage) was observed, except in the study by Sena et al. [110], who reported higher level of serum cobalt in lactating (56 ± 2 µg/100 mL) compared to pregnant (49 ± 3 µg/100 mL) and dried camels (23 ± 3 µg/100 mL). In a more recent study, with values within the range of 12.65–15.10 µg/100 mL, no significant differences were reported as an effect of the age, sex or even pregnancy status [147].

Cobalt supplementation in bolus form could improve the plasma Co status of camels [115]. A significant difference in cobalt blood status was observed between health camels and those affected by foreign bodies, at 30 ± 1.7 vs. 24.3 ± 2.0 µg/100 mL [148]. The blood cobalt also decreased in camels affected by anemia [96].

### 3.7. Iodine

Blood iodine is not currently used to assess the iodine intake in camels, contrary to the concentration of thyroid hormones, which is a more efficient way to assess the iodine status. Usually, the determination of iodine is performed in milk, because milk iodine is regarded as a better reflection of the status of the animal. It was suggested to determine plasma iodides in the blood [149], but to our knowledge there was no reference in camels. Regarding iodine, the rare values in camel reported in the literature are comparable to those for other herbivores, i.e., between 50 and 114 ng/mL [15].

The main symptom of iodine deficiency is obviously goiter, which has been observed in camels by several authors [150,151,152,153]. Most of the observed cases occurred in countries or regions far away from the coastal areas, where the iodine content in the pastoral resources is widely sufficient to meet the requirements of the herbivores [154]. In Saudi Arabia, several cases of goiter were described in different species, including in camels, with approximately 5% prevalence.

According to Abdel-Wahab and Osman [155], camels are more sensitive to iodine deficiencies compared to other animals due to their lower iodine absorption rate, but this fact was not confirmed by later studies. Moreover, a diet supplemented with potassium iodide can improve the milk production; with a 1 mg/kg DM intake of potassium iodide, the milk production was increased by 10% [156]. The effect of dehydration on the iodine status was investigated by several authors, showing a significant increase in blood iodine from 112 to 124 ng/mL after 10 days of water restriction [157]. Indeed, dehydration provokes a slowdown of the thyroid activity. This hypothyroidism leads to a reduction in iodine use by the thyroid gland, and generally to metabolism slowdown, which is a facet of the adaptation to water shortages for camels [158].

### 3.8. Other Elements

The other elements are less often investigated unless they attract local interest for specific deficiency and toxicity cases.

#### 3.8.1. Fluorine

For example, fluorine levels were determined in regions producing phosphates rocks (Morocco, Mauritania), which are known for their richness in fluorine and likely to cause fluorosis in grazing animals [159]. Some cases can also occur in industrial areas [160]. Due to its high calcium affinity, fluorine provokes modifications of the color and orientation of teeth and the structure and texture of bones [161], but with lower severity in camels than in sheep, cattle or even humans [162,163]. The normal level of plasma or serum fluorine in herbivores is generally below 30 µg/100 mL, but in camels living close to phosphate rocks in Morocco, the reported values appeared to be lower (6.4 ± 0.4 µg/100 mL and 4.5 ± 0.1 µg/100 mL in two localities), although they were higher than in remote areas of the phosphate mines (4.2 ± 0.1 µg/100 mL) [27]. Higher values were reported in cases of industrial contamination, for example in Egypt, at 190 ± 30 and 125 ± 7 µg/100 mL in male and female camels, respectively, in the more contaminated herds [161]. Some questionable values reported in Bactrian camels from China surpassed 1000 µg/100 mL, and even up to 2000 µg/100 mL [131].

#### 3.8.2. Molybdenum

Molybdenum is studied generally in cases of copper deficiency, because when it is associated with sulfur (S), it is an important antagonist of copper. The combination of Cu-Mo-S is not absorbable by grazing animals, leading to secondary copper deficiencies. The excess of molybdenum (molybdenosis) provokes acute diarrhea. This symptom was described in camels grazing on *Salvadora persica*, a bush growing in the volcanic soils of Djibouti and containing a high Mo content [80]. However, there was no relationship between the Mo status and reproduction disorders in male camels [164]. The blood values reported in the literature were 2.9 µg/100 mL [75] and 5.3 µg/100 mL [67] in dromedaries. In Bactrian camels, higher values were determined at 18 µg/100 mL [146], 19–23 µg/100 mL [25] and 43 µg/100 mL [141].

#### 3.8.3. Sulfur

Sulfur is usually considered an intermediate element between macronutrients and trace elements. In camels, the requirements for sulfur are not known, and the studies regarding this element are limited to the investigations of sulfur poisoning in cases of industrial pollution [160] or natural water wells in volcanic soils rich in sulfur [165]. Sulfur intoxication provokes emaciation, cachexia, respiratory distress, and polio-encephalitis. In intoxicated camels, the blood values reached 2085 ± 296 and 1882 ± 262 mg/100 mL in males and females, respectively, while the values reached 449 ± 52 and 503 ± 68 mg/100 mL in non-contaminated camels [160].

The Chinese references regarding Bactrian camels are comparable, at 412 ± 86 mg/100 mL in healthy camels and 631 ± 170 mg/100 mL in animals affected by ailments [146]. However, the chemical form of sulfur (organic or inorganic) was not differentiated in the above references.

#### 3.8.4. Bromide

Despite bromide not being an essential trace element, we mention it here because of its specific role in cases of dehydration. Indeed, bromide was determined in camel serum samples by Etzion et al. [157], reporting a value of 55.3 µg/mL. After 10 days of dehydration, this value increased up to 58.9 µg/mL, provoking sedation, contributing to metabolism slowdown and consequently participating in water preservation during water restriction.

#### 3.8.5. Nickel

Exposure to nickel is known to provoke DNA damage [166]. In camels, nickel was mentioned first in Mongolian Bactrian camels affected by Ni intoxication called “Roll disease” [167]. In dromedaries, Faye et al. [80] reported higher values in males (2 µg/100 mL) than in females (1.7 µg/100 mL).

## 4. Functional Indicators of Trace Element Status in Camels

Contrary to non-essential trace elements, the essential ones can lead to functional disorders in cases of failure (deficiency or excess). The use of supplementation can prevent or treat such disorder [28]. Moreover, the essential trace elements are organic molecules (enzymes, hormones, vitamins, metalloproteins) with important biological activity. These molecules are considered as functional indicators of the trace element status because any deficiency can impact their biological activity. In the case of metallo-enzymes, their activity is highly correlated with the trace elements present in their molecules.

The more studied metalloenzymes are ceruloplasmin (Cu), superoxide dismutase (Zn, Cu and Mn) and glutathione–peroxidase (Se). Some data regarding their values in camel blood are available [15].

### 4.1. Ceruloplasmin (Cp)

Cp contains six atoms of copper and carries 95% of the total copper present in the plasma. Camel Cp has been purified and characterized [168]. According to different authors, the Cp content range in the camel plasma is between 11 and 69 U/L [15]. It was reported that in cases of copper deficiency, the Cp activity is maintained. Therefore, while the relationship between Cp and blood copper is linear in cattle, a non-linear regression model appears more convenient in camels [169].

### 4.2. Superoxide Dismutase (SOD)

Different SOD molecules are trace-mineral-dependent; SOD1 and SOD3 are Zn/Cu-dependent, while SOD2 is Mn-dependent. Few references are available in camel whole blood [15], and the values varied between 1474 and 1813 IU/100gHb in a reference from Morocco [170] and between 1323 and 1412 IU/gHb in a reference from Iran [132]. Camel SOD was also recently purified [171].

### 4.3. Glutathione–Peroxidase (GSH-Px)

GSH-Px has been more widely studied than SOD in camels, and the values vary between 6.32 and 36 IU/g Hb in whole blood according to the different authors cited by [15]. The GSH-Px activity is sensitive to Se supplementation, and in camels this activity is maintained even after the end of the supplementation, contrary to in cows, probably in relation with the higher life expectancy of camel red cells [129]. In camels affected by selenosis, the GSH-Px value can reach 180 IU/g Hb [26]. As for the former metalloenzyme, camel GSH-Px was recently purified [172].

## 5. General Discussion

Due to limitations in the existing research and data regarding the requirements and functions of trace minerals for camels, the cattle data are currently used as a ruminant animal model, despite the physiological differences observed between camels and cattle [15,113], as well as between their environments [173]. Moreover, few studies have addressed the breed variability [89,138] or effects of the different feeding systems [35,90]. Seasonal variations were investigated in some studies (for example Khan et al. [174]), but such variability is generally the result of the seasonal changes in feeding resources.

### 5.1. Limiting Data and Constraints in Camel Trace Element Studies

Many studies regarding camels fail to consider the main factors that affect the mineral and nutritional status, such as the season, age, breed, sex, physiological status and management risk factors, especially those linked to the feeding system [15,173], irrespective of the substrate (meat, milk and plasma or serum, as well as tissues such as the liver, kidneys or muscles). Few studies have also focused on the potential link of the camel trace element status and soil or plant characteristics, although properties such as the clay content, content of organic matter and cation-exchange capacity of the soils can modify the absorption ability of the plants consumed by the animals [175]. Notably, two points must be emphasized regarding the impacts of trace element concentrations in other matrixes (soil, water and plants) on the animal status, namely the bioavailability of minerals (i.e., their chemical forms) and the interactions between minerals (for example, between copper, molybdenum and sulfur [80]). To our knowledge, these points have been rarely investigated in camel studies (for example, see the copper–zinc competition in camels in the study by Bengoumi et al. [170]). However, such aspects are not specific to camels.

Such information about the soil characteristics, bioavailability and competition between minerals can be helpful to establish a solid background for developing supplementation programs during different seasons to improve camels’ reproductive and production efficiency levels. When the trace elements are a limiting factor in the diet, supplementation improves the average daily gain, feed efficiency, liver mineral reserve and immune response [24].

However, it should be noted that under field conditions, the trace mineral status of animals in terms of their deficiency, adequate supply or excess is difficult to assess. For the majority of the biological materials that may be collected with reasonable effort (e.g., from the blood, milk, hair, feces or urine), the trace mineral concentrations overlap with the homeostatic regulation of the trace mineral metabolism, often with considerable delays in time (e.g., hair, erythrocytes). In trace minerals that are regulated mainly via urinary excretion (e.g., selenium, iodine), the concentrations in such biological materials may primarily reflect the respective dietary intakes rather than the supply status. Nevertheless, the trace mineral concentrations determined in such biological materials remain useful under field conditions, as they point at least to the direction of the supply status when collectively comparing data from an animal herd with the reported standard values. Until now, almost all scientific reports on the trace mineral supply of camels reflect such data derived under field conditions, mainly with blood serving as the primary biological material.

### 5.2. Camel Specificity

Camels live in relatively extreme environments providing more dietary minerals than the common range of habitats of other herbivores. Living in an environment with scarce resources, the camel has developed specific physiological mechanisms to adapt to such scarcity. These mechanisms include the management of the trace element metabolism to limit the biological effect of their deficit or excess in the milieu. These metabolic mechanisms include:

(1) The increased absorption of Cu and Zn under deficient conditions compared to cows [176];
(2) A higher accumulation capacity in cases of Cu deficiency compared to cows [111];
(3) The ability to tolerate excess minerals and electrolytes, as emphasized by the capacity to eat halophytes [159,177];
(4) The ability to better maintain the activities of key mineral-dependent enzymes compared to cows, such as GSH-Px [111], ceruloplasmin [169] or SOD [170].

Finally, the biology of camels appears as a physiological system able to anticipate the periods of resource restriction (water, food). This biological behavior with trace elements complies with the mechanisms of water preservation, urea recycling, fat storage management and the homeostasis of electrolytes, which result in the superiority of camels in harsh desert environments. However, despite these adaptations, camels in arid countries can suffer from a variety of mineral deficiencies, which are often undetected and untreated, resulting in significant economic losses, notably in intensive systems that are more demanding in terms of the production performance.

### 5.3. Gaps in Knowledge

Beyond the scarcity of data on the interactions with the statuses of other matrixes (soil, water, plants), many gaps in the knowledge still exist in relation to this species, such as (i) the lack of knowledge regarding the deficiency or toxicity of some elements; (ii) the requirements for new intensive systems, especially focused on dairy production; or (iii) the whole metabolism of some elements (ingestion, storage, excretion). Notably, a solid research program could be developed to achieve certain outcomes for a better understanding of (i) the effects of nutritional deficiencies or excesses on camels’ productivity and health; (ii) the interactions between mineral additives and supplements and meat or milk productivity; and (iii) the trace element requirements of camels according to their physiological status.

## 6. Conclusions

Camels live in environments richer in minerals compared to those of other domestic herbivores. However, they can be affected by mineral imbalances, even if their physiological ability to adapt in such harsh conditions can lead to a certain resistance to mineral deficiencies. The present review emphasizes the high variability observed in the serum and plasma concentrations according to the differences in physiological status, which is more rarely related to the environmental contexts (and probably to the analytical procedures). However, such values could support investigations on the trace element status or intake and could help clinicians in the interpretation of blood sampling results, as was shown in the present review regarding the zinc status in camels. However, further research is warranted for the elucidation of the requirements of this species in the context of the intensification and specialization of their production systems.

## Figures and Tables

**Table 1:** Comparative mean concentrations of copper in the plasma or serum of domestic ruminants (in μg/100 mL).

Country	Camel	Cattle	Sheep	Goat	Substrat	References
Egypt	83	64	82	NA	Serum	Moty et al. [75]
Sudan	95.3	73.8	85	78.9	Serum	Tartour [76]
Sudan	92.6	86.2	94.5	NA	Serum	AbuDamir et al. [77]
India	94.3	86.8	88.3	NA	Serum	Shekhawat [78]
Ethiopia	45	37.2	24.7	41.8	Plasma	Faye and Grillet [79]
Ethiopia	107	64.5	95.1	89.2	Plasma	Faye et al. [80]
Djibouti	60.7	73.8	87.2	94.5	Plasma	Faye et al. [81]
Saudi Arabia	113.5	70.2	95.6	NA	Serum	Al-Busadah [64]

NA: Non-Analyzed.

**Table 2:** Comparative mean concentrations of plasma or serum zinc in various species of domestic ruminants (in μg/100 mL).

Country	Camel	Cattle	Sheep	Goat	Substrate	Reference
Egypt	135	144	160	NA	Serum	Moty et al. [75]
India	85.4	86.8	94.8	NA	Serum	Shekhawat [78]
Ethiopia	100.4	113.5	114.2	107.7	Plasma	Faye et al. [62]
Djibouti	46.2	97.6	71.5	65.6	Plasma	Faye et al. [81]
Morocco	38	83	NA	NA	Plasma	Bengoumi et al. [111]
Saudi Arabia	103.4	98.5	110.7	NA	Serum	Al-Busadah [64]
Egypt	104.4	96.7	NA	NA	Serum	Khamis et al. [112]

NA: Non-Analyzed.

## Data Availability

Not applicable.
